# In Vitro Neurotoxicity of Flumethrin Pyrethroid on SH-SY5Y Neuroblastoma Cells: Apoptosis Associated with Oxidative Stress

**DOI:** 10.3390/toxics10030131

**Published:** 2022-03-07

**Authors:** Luis Barrios-Arpi, Yurie Arias, Bernardo Lopez-Torres, Mariella Ramos-Gonzalez, Giulio Ticli, Ennio Prosperi, José-Luis Rodríguez

**Affiliations:** 1 Animal Physiology Laboratory, Faculty of Veterinary Medicine, Major National University of San Marcos, Lima 41, Peru; lbarriosa@unmsm.edu.pe (L.B.-A.); yurie.arias@unmsm.du.pe (Y.A.); 2 Department of Toxicology and Pharmacology, Faculty of Veterinary Medicine, Complutense University of Madrid, 28040 Madrid, Spain; berlopez@ucm.es; 3 Zootecnia an Animal Production Laboratory, Faculty of Veterinary Medicine, Major National University of San Marcos, Lima 41, Peru; mramosgo@unmsm.edu.pe; 4 Institute of Molecular Genetics “Luigi Luca Cavalli-Sforza”, CNR, 27100 Pavia, Italy; giulio.ticli@igm.cnr.it (G.T.); ennio.prosperi@igm.cnr.it (E.P.); 5 Pharmacology and Toxicology Laboratory, Faculty of Veterinary Medicine, Major National University of San Marcos, Lima 41, Peru

**Keywords:** flumethrin, oxidative stress, apoptosis, neurotoxicity, SH-SY5Y cells

## Abstract

Pyrethroids are neurotoxicants for animals, showing a pattern of toxic action on the nervous system. Flumethrin, a synthetic pyrethroid, is used against ectoparasites in domestic animals, plants, and for public health. This compound has been shown to be highly toxic to bees, while its effects on other animals have been less investigated. However, in vitro studies to evaluate cytotoxicity are scarce, and the mechanisms associated with this effect at the molecular level are still unknown. This study aimed to investigate the oxidative stress and cell death induction in SH-SY5Y neuroblastoma cells in response to flumethrin exposure (1–1000 µM). Flumethrin induced a significant cytotoxic effect, as evaluated by MTT and LDH leakage assays, and produced an increase in the biomarkers of oxidative stress as reactive oxygen species and nitric oxide (ROS and NO) generation, malondialdehyde (MDA) concentration, and caspase-3 activity. In addition, flumethrin significantly increased apoptosis-related gene expressions (Bax, Casp-3, BNIP3, APAF1, and AKT1) and oxidative stress and antioxidative (NFκB and SOD2) mediators. The results demonstrated, by biochemical and gene expression assays, that flumethrin induces oxidative stress and apoptosis, which could cause DNA damage. Detailed knowledge obtained about these molecular changes could provide the basis for elucidating the molecular mechanisms of flumethrin-induced neurotoxicity.

## 1. Introduction

Many widely used pesticides, such as carbamates, organochlorine-phosphates, and others, have been withdrawn or are being discontinued due to their high levels of toxicity and their negative effects on the environment. Synthetic pyrethroids are used instead due to their selective toxicity to various types of insects, lower toxicity to mammals, and less detrimental effects on the environment [1]. Pyrethrum, a natural purified extract of *Chrysanthemum* spp. flowers, has been used as a natural insecticide for long-term exposure. The active components of pyrethrum are known as pyrethrins [2].

The main way to classify pyrethroids (Type I and II) is based on the presence of the α-cyano group in their chemical structure; for example, Type II has this group present in its chemical structure. The primary target of pyrethroids is the voltage-gated sodium channels present in the central nervous system [3,4]. In animals, Type I pyrethroids frequently produce T-syndrome (hyper-excitation and tremors), while Type II pyrethroids are associated with CS-syndrome (choreoathetosis and salivation) [5]. Globally, synthetic pyrethroids are widely used in the control of pests that infest agriculture, livestock, residences, and against vectors that affect public health [6,7].

Previous experiments have shown that pyrethroid exposure produces a greater effect in young animals [8], and several learning and memory-related changes have been reported in pyrethroid-treated rats, along with neurobehavioral changes that could be attributed to increased oxidative stress [9,10,11,12].

Flumethrin, a Type II synthetic pyrethroid, is commonly used against ectoparasites in several species such as bovines, sheep, goats, equines, and canines [6,13]. Flumethrin is constituted by more than 90% of the trans-Z-1 isomers, and [14] it does not absorb as quickly as other pyrethroids such as permethrin, deltamethrin, and λ-cyhalothrin [15]. Flumethrin has a long resistance time and half-life by oral and inhalation intoxication [15]. This pyrethroid is metabolized by central ester junction to form permethrin acid and 3-phenoxy-4-fluorobenzyl alcohol [13] and is principally excreted in the urine, and secondarily, in the feces. In vitro toxicity studies evaluating cytotoxicity, as well as the mechanisms at the molecular level associated with this effect, are still unknown.

The widespread use of pyrethroids has made human exposure and environmental contamination unavoidable. Therefore, further studies are needed to assess the neurotoxic effects of pyrethroids, which are of public health importance. Flumethrin is one of the most widely used Type II pyrethroid pesticides in various human activities [13], and studies describing the neurotoxic effects produced by this pyrethroid are still scarce [16]. In the present study, using SH-SY5Y cells, an in vitro model widely used for experimental studies in neurotoxicology [17], the possible association between oxidative stress, apoptosis, and flumethrin-induced neurotoxicity, was investigated. This in vitro study was performed to determine the concentration-dependent cytotoxicity of flumethrin using MTT (cell viability) and LDH assays to evaluate ROS and NO generation, MDA concentration, and caspase 3/7 activity, and to assess the expression of apoptotic genes (Bax, Bcl-2, Casp-3, BNIP3, AKT1 y APAF1) and oxidative stress and antioxidative genes (NFκB, NRF2, and SOD2) induced by flumethrin.

## 2. Materials and Methods

### 2.1. Chemicals and Reagents

Flumethrin, Cyano (4-fluoro-3-phenoxyphenyl)methyl3-[2-chloro-2-(4-chlorophenyl)ethenyl]-2,2-dimethylcyclopropanecarboxylate (CAS: 69770-45-2), ≥97% purity, molecular weight 510.38 g/mol; the compounds 2′,7′-dichlorofluorescin diacetate (DCFH), 4-amino-5-methylamino-2,7-difluorofluorescein diacetate (DAF-FM-DA), 3-[4,5 dimethylthiazol-2-yl]-2,5-diphenyl-tetrazolium bromide (MTT), malondialdehyde tetrabutylammonium salt (MDA), dimethyl sulfoxide (DMSO), Dulbecco’s phosphate buffered saline (DPBS, D8537), fetal bovine serum (FBS), and 2-thiobarbituric acid (TBA) were obtained from Sigma-Aldrich (St. Louis, MO, USA). Dulbecco’s Modified Eagle Medium/Nutrient Mixture F-12 (DMEM F-12) was obtained from BioWhittaker, Lonza (Walkersville, MD, USA), penicillin and streptomycin were obtained from Thermo Fisher (Waltham, MA, USA) and the Apo-ONE^®^ Homogeneous Caspase-3/7 Assay kit was acquired from Promega (Madison, WI, USA). All other chemical reagents used were of high purity for cell and molecular biology and were available in the laboratory.

### 2.2. Cell Line and Culture Condition

The SH-SY5Y (ATCC^®^ CRL-2266™) human neuroblastoma cell line (undifferentiated) was obtained from American Type Culture Collection (ATCC, Manassas, VA, USA). The SH-SY5Y cell line was cultured in a DMEM-F12 medium supplemented with heat-inactivated FBS (10%), to which 100 units/mL of penicillin and 100 µg/mL of streptomycin were added. Cultures were seeded in flasks containing the enriched medium and maintained at 37 °C in an environment with 5% CO_2_ and 95% oxygen. To carry out the tests, the cells were previously subcultured in 96-well plates at a seeding density of 5 × 10^4^ cells per well. Cells were treated with flumethrin (1–1000 µM, dissolved in DMSO) in DMEM-F12 (without phenol red) with 1% FBS for 24 h. A vehicle control group (0.1% DMSO) was also used in each experiment, and to maintain the physiological conditions of the SH-SY5Y cells, they were used with less than 13 passages.

### 2.3. Cell Viability (Cytotoxicity-MTT Assay)

The cell viability, or cytotoxicity, assay is based on the ability of mitochondrial activity to reduce the MTT reagent, which is determined by colorimetry [18]. Briefly, after flumethrin (1–1000 µM) exposure for 24 h, 50 μL of the MTT reagent (0.5 mg/mL, final concentration) were added to each well for 2 h, under the same incubation conditions (humidity, 37 °C, 5% CO_2_ and 95% oxygen). During these 2 h, metabolically active SH-SY5Y cells reduced the tetrazolium-MTT (yellow) to a formazan salt (purple). After 2 h, the insoluble formazan was dissolved with DMSO; the colorimetric determination of MTT reduction was measured in a microplate reader (ELx800, BioTek, Winooski, VT, USA) at 540 nm. The control group (with only the culture medium DMEM-F12) was considered to have 100% viability.

### 2.4. Lactate Dehydrogenase (LDH) Assay

The cytotoxic effect of flumethrin on the SHSY5Y cells was also determined by the LDH release from the affected cells into the extracellular fluid. LDH is a stable cytoplasmic enzyme present in all cells, which is rapidly released into cell culture supernatant after oxidative damage to the cell membrane. After flumethrin exposure for 24 h at several concentrations (1–1000 µM), samples were collected to estimate extracellular LDH as an indicator of cell death. LDH activity was measured spectrophotometrically using a Cytotoxicity Cell Death kit (Roche-Boehringer, Germany) according to the manufacturer’s indications. Total LDH activity was defined as the sum of intracellular and extracellular LDH activity, which was normalized as 100%; then, the amount of LDH released to the extracellular medium was expressed as a percentage of this total value. LDH activity was measured spectrophotometrically at 490–620 nm, using a microplate reader (ELx800, BioTek, Winooski, VT, USA).

### 2.5. Reactive Oxygen Species (ROS) Generation

Oxidative damage to cells, due to the effect of various chemical substances, can be assessed by the levels of cellular production of reactive oxygen species (ROS). ROS production was determined according to widely standardized protocols [19] by the 2′,7′-dichlorofluorescin diacetate (DCFH-DA) assay using a fluorescence microplate reader. DCFH-DA enters the cell and is oxidized by intracellular esterases that allows the release of DCFH (fluorescent compound). By quantifying the fluorescence, a fair estimate of the overall oxygen species generated under the different conditions was obtained. Briefly, DCFH-DA (10 µM, dissolved in DMSO) was added to each well (2 × 10^5^ cells/well) under similar incubation conditions (humidity, 37 °C, 5% CO_2_, and 95% oxygen) in a black multiwell plate for 30 min. Then, the cells were washed twice with DPBS, and a culture medium with treatments (1–1000 µM flumethrin) was added to each well. The multiwell plates were immediately measured using a fluorescent microplate reader (FLx800 Fluorimeter, BioTek, Winooski, VT, USA) at an excitation/emission wavelength (485/530), measured innm.

### 2.6. Nitric Oxide (NO) Production

NO is a messenger that signals multiple intracellular and intercellular physiological processes [20]. Changes in NO secretion were determined by direct measurement using the DAF-FM-DA assay. Briefly, for the assay of the direct effect of flumethrin, cells were seeded in 96 black multiwell plates at a rate of 8 × 10^4^ cells. After a 24 h incubation period, 1 µL DAF-FM-DA (1 mM DAF-FM-DA stock solution was added to each well assay to obtain a final concentration of 10 μM) was added and the cells were incubated for 30 min at 37 °C. Then, the cells were washed twice with DPBS. SH-SY5Y cells were incubated with flumethrin (1–1000 µM) for 30 min at 37 °C. The concentration of nitric oxide was measured by fluorimetry (FLx800 Fluorimeter, BioTek, Winooski, VT, USA) at an excitation/emission wavelength (495/515), measured in nm.

### 2.7. Lipid Peroxidation Determination

Malondialdehyde (MDA), is an important indicator of the oxidative damage suffered when cell membranes are senescence, and is one of the toxic substances produced by the increase in free radicals. MDA has a high capacity to produce cell alteration due to its high reaction capacity with biological macromolecules such as proteins and nucleic acids. Furthermore, MDA has the ability to change the selectivity and permeability of cell membranes and thus cell structure/function. We evaluated the concentration of MDA induced by flumethrin (1–1000 μM) after the 24 h incubation period. The MDA levels were determined using a thiobarbituric acid reagent-based assay (TBARS) (Cell Biolabs, San Diego, CA, USA) according to the manufacturer’s protocol. Briefly, 1 × 10^6^ cells per well were seeded in a six-well plate; then, cells were collected in 200 μL of culture medium and sonicated for 3 × 5 s intervals at 40 V over ice. Next, SDS lysis solution (100 μL) was added to the sample solution and the MDA standards in a microcentrifuge tube and mixed well. Subsequently, 250 μL of TBA reagent were added to the samples and incubate at 95 °C for 60 min. Each sample and standard (200 μL) was transferred in triplicate into a clear 96-well plate and the absorbance at 532 nm was recorded using a microplate reader (ELx800 BioTek, Winooski, VT, USA). Finally, the concentration of MDA for each sample was determined from a standard curve.

### 2.8. Caspase 3/7 Activity-Fluorescence Assay

The Apo-ONE^®^ Homogeneous Caspase-3/7 Assay (Promega, Madison, WI, USA) is a fluorescent assay that measures caspase-3 and -7 activities homogeneously and includes a profluorescent substrate with an enhanced bifunctional cell lysis buffer for caspase-3/7 activity assays. Caspase-3/7 rhodamine 110 substrate, bis-(N-CBZ-L-aspartyl-L-glutamyl-L-valyl-L-aspartic acid amide) (Z-DEVD-R110) is the fluorescent substrate used in this study. To perform the Apo-ONE assay, the buffer and substrate were mixed and added to each sample. After cleavage and removal of DEVD peptides due to caspase-3/7 activity (excitation wavelength 499 nm), rhodamine 110 becomes highly fluorescent (emission wavelength 521 nm). The magnitude of fluorescence generated is proportional to the caspase-3/7 cleavage of the sample.

The SH-SY5Y cell line (15 × 10^3^ cells/well) was grown in black 96-well plates and exposed to flumethrin (1–1000 μM) for 24 h. After treatment, the Apo-ONE^®^ Caspase-3/7 Assay was prepared according to the manufacturer’s instructions. We removed the 96-well plates containing the treated cells from the incubator, and 100 µL of homogeneous Caspase-3/7 reagent was added to 100 µL of the culture medium containing the previously treated cells in each well and incubated at room temperature for 60 min in the dark. Fluorescence (excitation/emission wavelength 485/528 nm) was measured using the plate reader (FLx800, BioTek, Winooski, VT, USA). Data were normalized as % control.

### 2.9. RNA Extraction/Purification, cDNA Synthesis and Real-Time PCR

The SH-SY5Y cell line was treated with flumethrin (20, 50, and 500 μM) for 24 h. Total RNA was obtained using the TRIzol Reagent method (Thermo Fisher, Waltham, MA, USA) and purified using the RNeasy MinElute Cleanup Kit according to the manufacturer’s instructions (Qiagen, Germantown, MD, USA). The total RNA concentration and purity was quantified using a Nabi UV/Vis Nano Spectrophotometer (Microdigital, Seoul, Kr, Korea), obtaining A260/A280 ratios > 1.9, 2.1< in all the samples. The cDNA was synthesized from 2 μg of total RNA by reverse transcription (RT) using the RT2 First Strand kit (Qiagen, Germantown, MD, USA) according to the manufacturer’s instructions, beginning with a genomic DNA removal step. Finally, the cDNA was diluted 1:10 in nuclease-free water and stored at −80 °C for further analysis. Quantitative real-time PCR assays for Bax, Casp-3, Bcl-2, BNIP3, AKT1, APAF1, NFκB, NFR2, and SOD2 genes linked to apoptosis and oxidative stress-antioxidant mechanism were performed to analyze mRNA gene expressions. The cDNA amplification was performed using a real-time PCR system (BioRad CFX, Hercules, CA, USA), using RT2 SYBR Green qPCR Mastermix (Qiagen, Germantown, MD, USA), according to the manufacturer’s instructions. For RT-PCR, it was necessary to use primers with concentrations of 400 nM, and the thermocycling protocol was programmed at 95 °C for 10 min, followed by 40 cycles of 15 s at 95 °C, and 1 min at 60 °C. Forward and reverse primers are presented in Table 1. Relative changes in gene expression were calculated according to Pfaffl (2001) [21], using glyceraldehyde-3-phosphate dehydrogenase (GAPDH) as a housekeeping gene and extracting the efficiencies from raw data using LinRegPCR software [22].

### 2.10. Statistical Analysis

All tests were performed in triplicate with six independent experiments and the results were represented as mean value ±standard deviation (SD). Statistical comparison between experimental and control groups was performed by one-way analysis of variance (ANOVA), powered by Tukey’s post-hoc test, using GraphPad Prism 7 software. The statistical difference found between groups was significant from *p* < 0.05. IC_50_; value was determined by the concentration-response curve using OriginPro 9 software.

## 3. Results

### 3.1. Flumethrin Reduced Cell Viability (Cytotoxicity)

**Figure 1:**
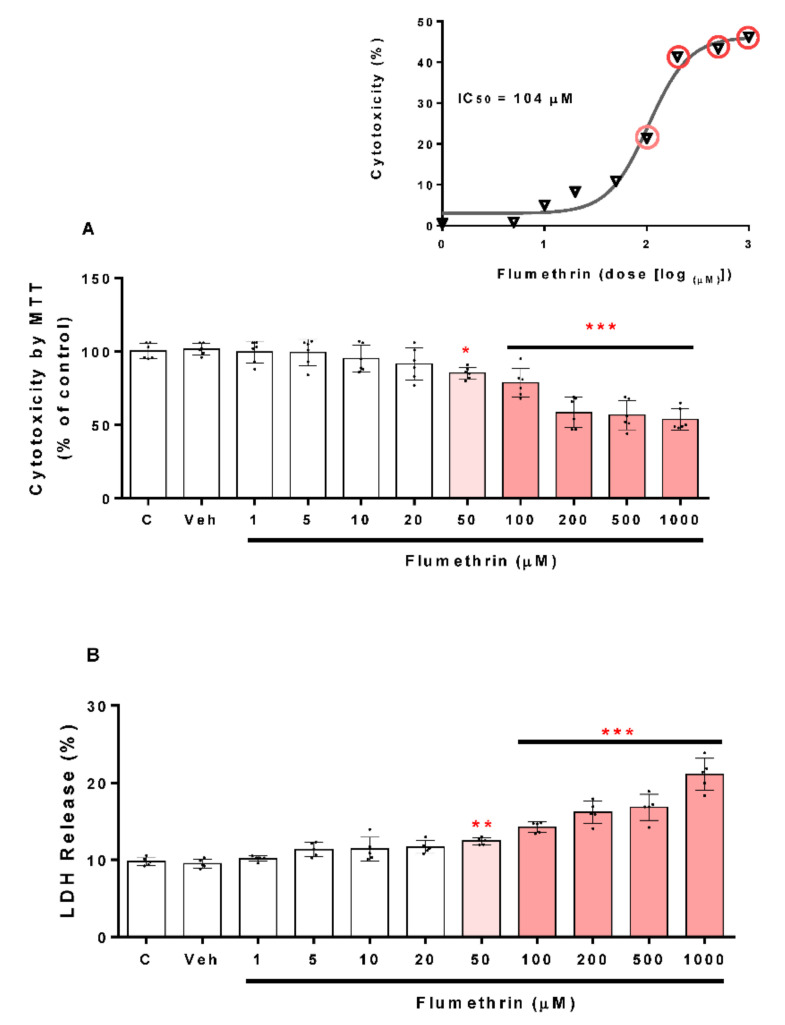
The cytotoxicity induced by flumethrin (1–1000 µM) on the viability of the SH-SY5Y cell line after a 24 h incubation period. Cell viability was determined by MTT reduction, and the MTT reduction (%) dose-response curve was used to obtain the IC_50_ value (**A**), or as LDH release (**B**). Data was normalized as % control (c). DMEM-treated cells with 1% FBS were the positive control (c), and cells with 0.1% DMSO were the negative control (Veh). Results are presented as the mean ± SD of six replicates. * *p* < 0.05, ** *p* < 0.01, *** *p* < 0.001 compared to Veh.

To assess the cell viability of the SH-SY5Y cells, we used the MTT assay (described above). Figure 1A shows the statistically significant difference found between the groups treated with flumethrin versus the control group (Veh, 0.1% DMSO). Incubation for 24 h with flumethrin at increasing concentrations (50, 100, 200, 500, and 1000 μM) reduced cell viability (15%, 22%, 41%, 43%, and 47%, respectively) in a concentration-dependent manner when compared with vehicle-treated cells (Veh, negative control). The IC_50_ value for flumethrin was calculated to be 104 μM (Figure 1A). We also examined this cytotoxic effect of flumethrin by assessing LDH release. The SH-SY5Y cells incubated for a 24 h period with flumethrin at the doses of 50, 100, 200, 500, and 1000 μM produced significant elevations in LDH leakage (24%, 42%, 61%, 68%, and 110%, respectively) (Figure 1B) compared to vehicle-treated cells (Veh, negative control).

### 3.2. Flumethrin Induces Oxidative Stress through the Production of ROS, NO, and MDA Levels

**Figure 2:**
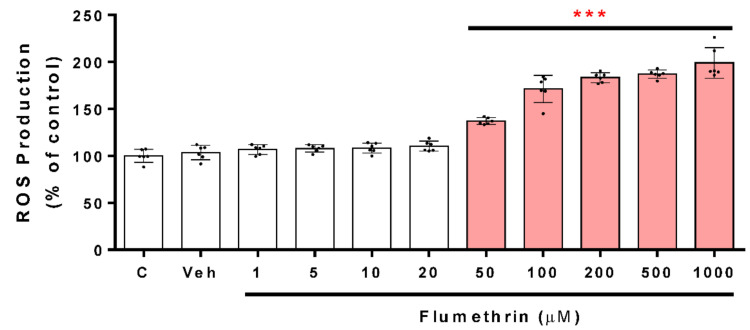
The ROS production induced by flumethrin (1–1000 µM) in SH-SY5Y cells after a 24 h incubation period. ROS production is expressed as % of control. DMEM-treated cells with 1% FBS were the positive control (c), and cells with 0.1% DMSO were the negative control (Veh). Results are presented as the mean ± SD of six replicates. *** *p* < 0.001 compared to Veh.

This assay was made in order to evaluate an imbalance in the oxidative status caused by the effect of flumethrin on neuroblastoma cells SH-SY5Y; ROS generation was measured. Flumethrin at concentrations from 50 to 1000 μM induced a dose-dependent increase in ROS generation. Flumethrin at concentrations of 50, 100, 200, 500, and 1000 μM significantly (*p* < 0.001) induced an increase in ROS by 37%, 71%, 83%, 87%, and 99%, respectively, with respect to the control (Figure 2).

**Figure 3:**
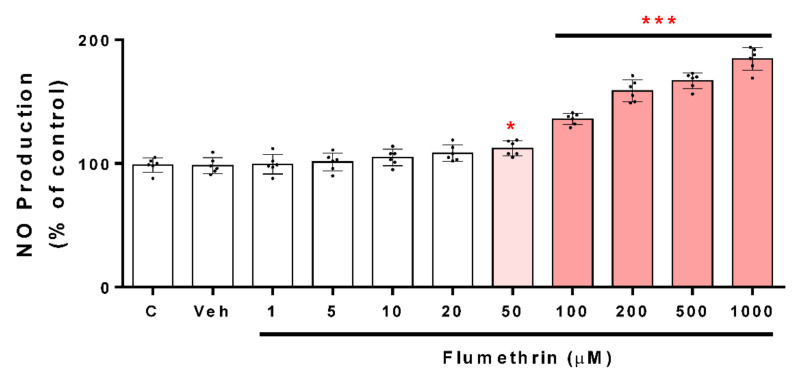
The NO production induced by flumethrin (1–1000 µM) in SH-SY5Y cells after a 24 h incubation period. Data was normalized as % control. DMEM-treated cells with 1% FBS were the positive control (c), and cells with 0.1% DMSO were the negative control (Veh). Results are presented as the mean ± SD of six replicates. * *p* < 0.05, *** *p* < 0.001 compared to Veh.

**Figure 4:**
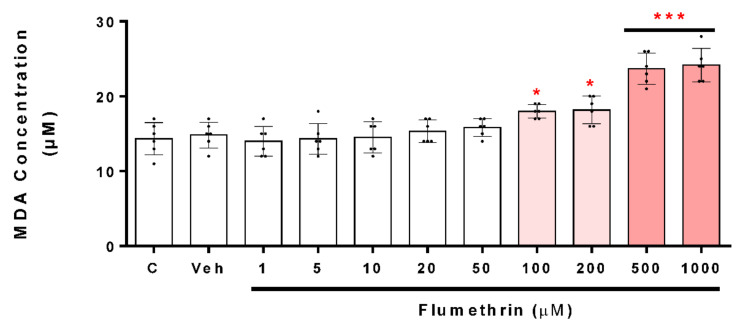
The MDA production induced by flumethrin (1–1000 µM) in SH-SY5Y cells after a 24 h incubation period. The content of MDA (µM) was calculated for each sample from a standard curve. DMEM-treated cells with 1% FBS were the positive control (c), and cells with 0.1% DMSO were the negative control (Veh). Results are presented as the mean ± SD of six replicates. * *p* < 0.05, *** *p* < 0.001 compared to Veh.

A significant increase in intracellular NO could indicate alterations in cellular health. Furthermore, an excess of NO can initiate a neurotoxic cascade. In Figure 3 and Figure 4, neuroblastoma cells exposed to flumethrin (50 to 1000 μM) after a 24 h incubation period, produced, in a dose-dependent manner, a significant increase in NO production. The 50 μM dose of flumethrin increased the NO generation by 12% (*p* < 0.05) with respect to the control, while the doses of 100, 200, 500, and 1000 µM produced a significant (*p* < 0.001) increase of 36%, 59%, 67%, and 84%, respectively, with respect to the control (Figure 3).

Lipid peroxidation is the oxidative degradation reaction of lipids, and MDA is its main intermediate product. Figure 4 shows the MDA concentration induced by flumethrin (from 100 to 1000 µM) after a 24 h incubation period. Flumethrin (100 μM) was the lowest concentration that caused a significant increase in MDA levels (28%, *p* < 0.05 compared to control). Incubation with flumethrin (1000 μM) induced a significant increase in MDA levels (166%, *p* < 0.001 compared to Veh)

### 3.3. Flumethrin Induces the Caspase 3/7 Activity, an Apoptosis Mediator

**Figure 5:**
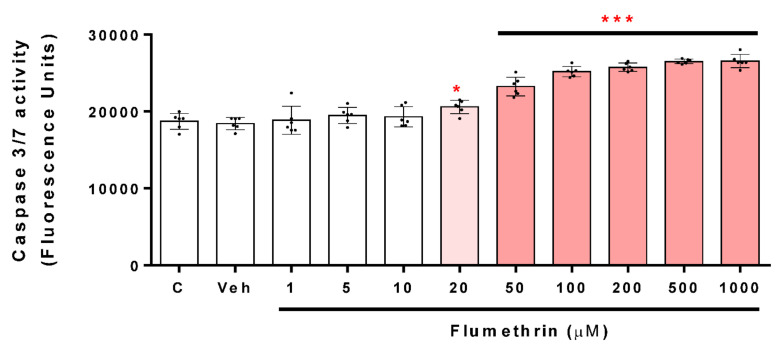
The caspase 3/7 activity induced by flumethrin (1–1000 µM) in SH-SY5Y cells after a 24 h incubation period. DMEM-treated cells with DMEM with 1% FBS were the positive control (c), and cells with 0.1% DMSO were the negative control (Veh). Results are presented as the mean ± SD of six replicates. * *p* < 0.05, *** *p* < 0.001 compared to Veh.

Caspase 3/7 activation is a critical part of apoptosis; the reagent used in this study was optimized for apoptosis analysis. Our work shows that caspase 3/7 activity was also increased in the treated cells compared to the vehicle-group cells after a 24 h incubation period. Flumethrin concentrations of 20, 50, 100, 200, 500, and 1000 cause an increase in caspase 3/7 activity of 10% (*p* < 0.05), 24%, 34%, 37%, 42 and 42% (*p* < 0.001), respectively, with respect to the control (Figure 5).

### 3.4. Flumethrin Effect on Apoptosis and Oxidative Stress and Antioxidative Gene Transcriptions in the SH-SY5Y Cell Line

**Figure 6:**
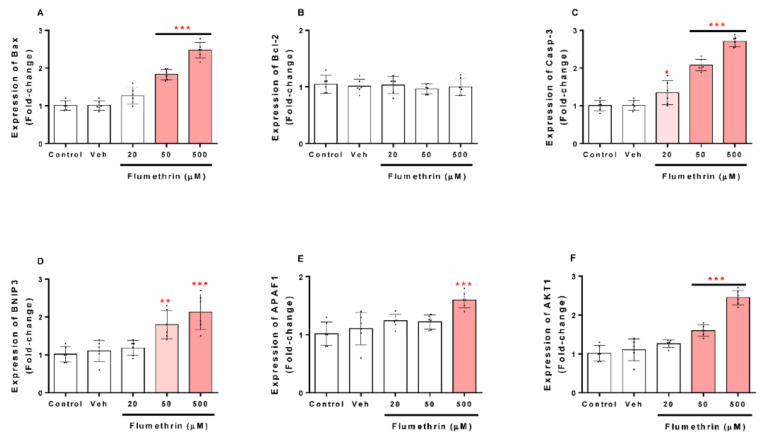
The effect of flumethrin (20, 50, and 500 µM) in SH-SY5Y cells after a 24 h incubation period on Bax (**A**), Bcl-2 (**B**), Casp-3 (**C**), BNIP3 (**D**), APAF1 (**E**), and AKT1 (**F**) gene expressions related to apoptosis. All data were normalized with GAPDH expression and presented as relative to control. Data are expressed as fold-change with respect to the control (c). Cells with 0.1% DMSO were the negative control (Veh). Results are presented as the mean ± SD of four replicates. * *p* < 0.05, ** *p* < 0.01 *** *p* < 0.001 compared to vehicle.

Regarding the effects of flumethrin on apoptosis-related gene transcription in the SH-SY5Y cell line, Bax, Casp-3, BNIP3, APAF1, and AKT1 mRNA levels were increased after flumethrin exposure (20, 50, and 500 μM) (Figure 6A,C–F). Flumethrin at a 500 µM dose produced the most significant (*p* < 0.001) increase in mRNA levels observed for Casp-3 (2.7-fold), Bax (2.5-fold), and AKT1 (2.5-fold), followed by BNIP3 (2.1-fold) and APAF1 (1.6-fold). In addition, flumethrin at a 50 µM dose induced Casp-3 (2.1-fold, *p* < 0.001), Bax (1.8-fold, *p* < 0.001), BNIP3 (1.8-fold, *p* < 0.01), and AKT1 (1.6-fold, *p* < 0.001) mRNA expression. However, at a 20 µM dose, only Caps-3 mRNA was increased (1.3-fold, *p* < 0.05).

**Figure 7:**
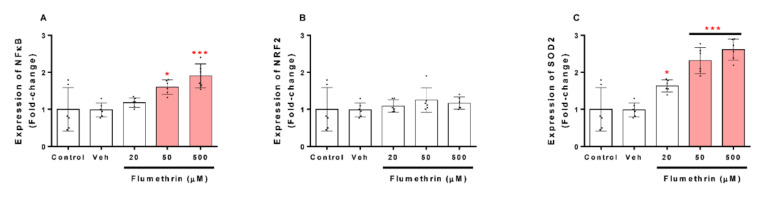
The effect of flumethrin (20, 50, and 500 µM) in SH-SY5Y cells after a 24 h incubation period on NFκB (**A**), NRF2 (**B**), and SOD2 (**C**) gene expressions related to oxidative stress and the antioxidant gene transcription. All data were normalized with GAPDH expression and given as relative to control. Data are expressed as fold-change with respect to the control (c). Cells with 0.1% DMSO were the negative control (Veh). Results are presented as the mean ± SD of four replicates. * *p* < 0.05, *** *p* < 0.001 compared to vehicle.

Regarding the effects of flumethrin on oxidative stress and the antioxidant system transcription related to SH-SY5Y, our study demonstrated that NFkB and SOD2 mRNA expression increased after flumethrin exposure (20, 50, and 500 μM) (Figure 7). A significant increase in mRNA levels was observed for SOD2 at 20 µM (1.6-fold, *p* < 0.05), 50 µM (2.3-fold, *p* < 0.001), and 500 µM (2.6-fold, *p* < 0.001) of flumethrin (Figure 7B) and for NFkB to 50 µM (1.6-fold, *p* < 0.05) and 500 µM (1.9-fold, *p* < 0.001) of flumethrin (Figure 7C). Another gene related to oxidative stress that we evaluated was NRF2, but there was no significant increase in this gene due to the effect of pyrethroid flumethrin.

## 4. Discussion

Flumethrin is a Type II synthetic pyrethroid with a wide spectrum of insecticidal and acaricidal activity; it is used to control a wide range of insects such as ticks, mites, and fleas [16,23,24]. This pyrethroid exerts its neurodegenerative effects by causing disorders of the central nervous system [16]. Fewer studies reveal the neurotoxicity of flumethrin in vitro than in vivo. However, very little known about its effects on biological pathways, such as oxidative stress, inflammation, and programmed cell death. To our knowledge, this is the first study to reveal the effects of flumethrin on two processes: oxidative stress and cell death (apoptosis). This particular in vitro study using the SH-SY5Y neuroblastoma cell line indicates new mechanisms that could be involved in flumethrin-induced neurotoxicity. In fact, our work reveals that pyrethroid flumethrin, at concentrations below those that produce clinical signs of neurotoxicity, may have overt deleterious effects on the cells of the nervous system.

The cytotoxic effect of flumethrin occurred at the concentration of 50 μM. The IC_50_ value for flumethrin (104 µM) was higher than that found for other pyrethroids such as alpha-cypermethrin (78.3 μM) [25] and cyfluthrin (19.11 μM) [26], indicating a lower cytotoxic effect of flumethrin on SH-SY5Y cells as compared to other synthetic pyrethroids [27]. These results were confirmed by the LDH assay, and similar results were observed using the pyrethroid cypermethrin in SH-SY5Y cells [25].

Oxidative stress has been postulated to be one of the most important issues in insecticide toxicology [25,28,29,30]. Several in vitro studies have shown the potential ability of pyrethroids to produce oxidative stress [25,26,31,32,33]. The present study showed that SH-SY5Y neuroblastoma cells treated with flumethrin (1–1000 μM) in a dose-dependent manner produced a significant cellular ROS formation. ROS are produced by cellular metabolic reactions and when these reactions exceed physiological and antioxidants defenses, they could be implicated in the pathogenesis of several diseases, including atherosclerosis, cancer, amyotrophic lateral sclerosis, as well as Parkinson’s and Alzheimer’s disease [34,35,36]. ROS are highly reactive and harmful because they alter the structure and function of all cellular macromolecules, producing behavioral abnormalities, cytotoxicity, and even mutagenic damage. Furthermore, it has been proposed that dopaminergic neurons, such as the SH-SY5Y cell line, are more susceptible to damage caused by ROS. Our study found that SH-SY5Y neuroblastoma cells exposed to flumethrin (at 50 μM, the lowest effective dose) produced a significant increase in ROS (37% with respect to the control). Martínez et al. (2020) [33] reported that the 2.5 μM dose of cyfluthrin and the 5 μM dose of alpha-cypermethrin increased ROS generation by 22% and 24%, respectively. This marked difference could be related to a less toxic effect of flumethrin, 20 times less than cyfluthrin and 10-fold less than cypermethrin. The increase in ROS production is a biomarker of oxidative stress and may represent one of the mechanisms by which pyrethroids such as flumethrin, cyfluthrin, cypermethrin, or other pyrethroids damage the brain and represent risk factors for neurodegenerative diseases [37].

Exposure to insecticides such as pyrethroids can induce the overproduction of nitrogen-related free radicals (reactive nitrogen species, RNS) [38]. When NO reacts with the superoxide radical (O_2_^−^), the production of intracellular and extracellular peroxynitrite (ONOOH-) is induced. ONOOH- in the presence of hydrogen ions can produce hydroxyl radicals (HO•), powerful oxidizing agents that induce damage to several biomolecules, such as DNA and proteins [39]. In this study, we show that SH-SY5Y cells treated with flumethrin (50, 100, 200, 500, and 1000 µM) in a dose-dependent manner produced an increase in NO levels (12%, 36%, 59%, 67%, and 84%, respectively, with respect to the control). Therefore, the increase in NO suggests that flumethrin induces a greater synthesis of superoxide radical, with a consequent induction of changes in antioxidant enzymes and energetic reserves. Romero et al. (2017) [25] showed that alpha-cypermethrin at 60 µM dose increased NO production by 250% in the SH-SY5Y cell line, and Martinez et al. (2019) [26] reported that cyfluthrin increased NO generation by 100% in SH-SY5Y cells; in another in vivo study, cypermethrin (80 mg/kg bw) increased NO levels by 42% in a rat’s brain [40].

An increase in free radicals such as ROS and RNS can generate the lipid peroxidation process in an organism, and MDA is one of the final products of polyunsaturated fatty acids peroxidation in cells. Therefore, the overproduction of free radicals increases MDA levels, which is considered as an index of oxidative stress [41]. In this study, MDA levels were elevated from the 100 µM dose (28% with respect to the control). The high levels of MDA would be a consequence of the high production of ROS and other free radicals induced by flumethrin. This same effect has been observed for cyfluthrin and cypermethrin pyrethroids on SH-SY5Y cells, in humans as well as in rats [25,26,40].

ROS induces cell apoptosis by releasing apoptogenic proteins such as cytochrome c [42], which activates caspase-9, and in turn, cleaves and activates caspase-3. Once caspase-3 is activated, several specific substrates for caspase-3 are cleaved, ultimately leading the cell to apoptosis [43]. Flumethrin produced an increase in ROS and other oxidative mediators in SH-SY5Y cells that could be involved in caspase-3 activation. We show that caspase 3/7 activity was significant for the effect of flumethrin at 20 (10%), 50 (24%), 100 (34%), 200 (37%), 500 (42%), and 1000 (42%) µM doses. A study reported by Martínez et al. (2020) [33] showed that cypermethrin (25 µM) and cyfluthrin (2.5 µM) induced caspase 3/7 activity by 22% and 24% relative to the control, respectively. Furthermore, other non-pyrethroid pesticides such as glyphosate (5 mM, 71%) and its metabolite (10 mM, 78%) produced increased caspase 3/7 activity in SH-SY5Y cells [44]. These effects confirm that pyrethroid and non-pyrethroid pesticides at low doses are capable of producing apoptosis that could induce neurodegeneration.

The SH-SY5Y cells treated with flumethrin had a fold-change greater than 1.5 compared to the control in Bax, Casp-3, BNIP3, APAF1, and AKT1 expression, which were involved in cell death processes. These findings allow us to demonstrate that flumethrin may exert cell death effects involving apoptosis. These results are comparable with other studies that evaluated the effects of alpha-cypermethrin and cyfluthrin on the expression of a wide variety of pro-apoptotic molecules including caspase-3 and Bax, important endogenous regulators of cellular activity in response to a wide variety of physiological and pathological injuries [25,26,33,45].

Bcl-2 is a family of proteins that possess either proapoptotic (Bax) or antiapoptotic (Bcl-2) properties [46]. Romero et al. (2017) [25] revealed that alpha-cypermethrin produced an increase in caspase-3 and Bax; however, they showed that this pyrethroid also increased Bcl-2 levels. Other authors, such as Martinez et al. (2019, 2020) [26,33], showed similar results. Our study showed that flumethrin had no effect on the Bcl-2 levels in neuroblastoma cells. As described above, flumethrin exposure (20, 50, and 500 µM) induced apoptotic cell death in SH-SY5Y cells by the upregulation of caspase-3 (1.3, 2.1, and 2.7-fold, respectively, compared to the control), which means that an extrinsic apoptosis pathway was activated by this pyrethroid [47,48]. Caspase-3 is a downstream effector of caspase-9, and it plays a critical role in apoptosis [49].

The present study showed, through real-time PCR assays, that flumethrin (50 and 500 µM) produced an upregulation of three genes involved in the apoptosis processes. The genes upregulated by flumethrin included BNIP3 (1.8 and 2.1-fold), AKT1 (1.6 and 2.5-fold), and APAF1 (1.6-fold, only at 50 µM). These results are comparable with of other studies that evaluated the effects of alpha-cypermethrin on the expression of a wide variety of pro-apoptotic molecules including AKT1 and APAF1, important signals of cellular activity in response to a wide variety of physiological and pathological injuries. BNIP3 is considered to have the function of pro-apoptosis. The overexpression of BNIP3, a mitochondrial pro-apoptotic protein and a mediator of hypoxia-induced cell death [50,51], could also suggest that the flumethrin-induced oxidative stress could be mediated by this BNIP3 activation. In the present study, SH-SY5Y neuroblastoma cells treated with flumethrin (50 and 500 µM) produced an increase in the expression of this gene (1.8 and 2.1-fold). This result is similar to those reported by Martinez et al. (2020) [33], but differs from those reported by Martinez et al. (2019) [26], who found a 7-fold increase similar to caspase-3, demonstrating differences in the ROS-inducible effect between pyrethroids of the same type (cyfluthrin against flumethrin).

AKT1 is considered as a mediator of the phosphoinositide signaling, and its activation generates phosphorylation of several cellular proteins that are involved in the processes of cellular metabolism, proliferation, and apoptosis of nervous system cells [52]. Flumethrin could induce ROS production through the AKT1 pathway. In this study, flumethrin (50 and 500 µM) produced an increase in this molecule (1.6 and 2.5-fold) in SH-Y5Y neuroblastoma cells. This result is higher than reported by Romero et al. (2017) [25], who evaluated the effect of α-cypermethrin (60 µM) in the same type of neuronal cells. In their study, cyfluthrin (5 µM) showed an 8-fold increase in AKT1, demonstrating the lower flumethrin-inducing effect of certain apoptosis molecules when compared to other pyrethroids [26]. Furthermore, flumethrin (500 µM) induced the expression of APAF1 (1.6-fold), a protein that activates one of the important regulators of apoptosis, caspase-3 [53], suggesting that the apoptosome activation effect of flumethrin is lower than that of other pyrethroids [25,26,33].

Finally, we have been able to demonstrate the relationship between oxidative stress and apoptosis, closely associated events that have already been demonstrated in other pyrethroid studies [54,55]. Our findings on the gene expression of NFκB and SOD2 show that flumethrin (50 and 500 µM) is able to induce the mRNA of these two molecules involved in the oxidative and antioxidative state, which is confirmed by the findings of additional studies on pyrethroids and other pesticides [26,56].

## 5. Conclusions

Overall, the present study intends to include new data that suggest that the association between oxidative stress and apoptosis plays an essential role in the neurotoxicity produced by the pyrethroid flumethrin in SH-SY5Y cells. However, flumethrin appears to be less toxic than other pyrethroids tested on SH-SY5Y cells, but that does not mean that it is not toxic under frequent or long-term use. Our study provides relevant information on the possible mechanisms of cytotoxicity in SH-SY5Y cells after exposure to flumethrin, such as the increased production of ROS, NO, MDA, and caspase 3/7 activity, and the overexpression of apoptotic (Bax, Casp-3, BNIP3, AKT1, and APAF1) and oxidative stress and antioxidative (NFκB and SOD2) genes. Furthermore, additional information is needed using this model in vitro, or using primary human neuronal cells cultures that allow an approach to in vivo neurotoxicology studies, and involving more genes to better understand the effects of flumethrin from the point of view of neurodegeneration.

## Figures and Tables

**Table 1:** The sequences of forward and reverse primers for oxidative stress and apoptosis related genes.

Genes	Primer Forward Sequence	Primer Reverse Sequence
Housekeeping gene		
GAPDH	GAGAAGGCTGGGGCTCATTT	AGTGATGGCATGGACTGTGG
Apoptosis related genes		
Bax	CCCCCGAGAGGTCTTTTTCC	CCTTGAGCACCAGTTTGCTG
Casp-3	GTGGAGGCCGACTTCTTGTA	TTTCAGCATGGCACAAAGCG
Bcl-2	TCTCATGCCAAGGGGGAAAC	TCCCGGTTATCGTACCCTGT
BNIP3	CCTCAGCATGAGGAACACGA	GCCACCCCAGGATCTAACAG
AKT1	GAAGGACGGGAGCAGGC	TGTACTCCCCTCGTTTGTGC
APAF1	TCTTCCAGTGGTAAAGATTCAGTT	CGGAGACGGTCTTTAGCCTC
Oxidative stress and antioxidative related genes		
NFκB1	TTTTCGACTACGCGGTGACA	GTTACCCAAGCGGTCCAGAA
Nrf2	CTGGTCATCGGAAAACCCCA	TCTGCAATTCTGAGCAGCCA
SOD2	CCACTGCTGGGGATTGATGT	CGTGGTTTACTTTTTGCAAGCC

## Data Availability

Not applicable.
